# The topology of between-herd cattle contacts in a mixed farming production system in western Kenya

**DOI:** 10.1016/j.prevetmed.2018.06.010

**Published:** 2018-10-01

**Authors:** J. Ogola, E.M. Fèvre, G.K. Gitau, R. Christley, G. Muchemi, W.A. de Glanville

**Affiliations:** a Department of Public Health Pharmacology and Toxicology, University of Nairobi, PO Box 9053-00625, Nairobi, Kenya; b International Livestock Research Institute, PO Box 30709-00100, Nairobi, Kenya; c Centre for Infection, Immunity & Evolution, School of Biological Sciences, University of Edinburgh, Ashworth Laboratories, Kings Buildings, West Mains Rd, Edinburgh, EH9 3JT, UK; d Department of Clinical Studies, University of Nairobi, PO Box 29053-00625 Nairobi, Kenya; e Institute of Infection and Global Health, University of Liverpool, Leahurst Campus, Neston, CH64 7TE, UK

**Keywords:** Topology, Heterogeneity, Networks, Smallholder

## Abstract

In many livestock production systems in sub-Saharan Africa, cattle are owned by individual keepers but regularly mix with animals from other herds while grazing communal land, at watering points or through the use of shared bulls for breeding and ploughing. Such contacts may have important implications for disease transmission and control but are not well documented.

We describe between-farm contacts in Kimilili sub-county of Bungoma County, a mixed farming area of predominately smallholder farmers. Between-farm contacts occurring during grazing or at shared water points over the past four weeks were captured in seven randomly selected villages using a photo-elicitation tool. The use of shared bulls for breeding and ploughing and cattle introductions from farms within the same village in the past 12 months were also captured. Contact networks were constructed for each contact type in each village.

In total 329 farms were included in the study. Networks resembled undirected scale-free graphs with a network density ranging between 9.6 and 14.0. Between 45.6 and 100% of the farms in each study village had been in contact over the past four weeks through grazing and watering contacts. Between 88.9 and 100% were considered to have been in contact over the past 12 months. The topology of the networks was heterogeneous, with some farms exhibiting a high degree of contact. The degree of farm contact and distances between farms were negatively correlated (Pearson correlation coefficient range −0.2 to −0.4).

Effective disease control and surveillance must take into consideration the frequency and range of contacts that occur between farms within a single village. Cattle keepers are highly interconnected and pathogens that are transmitted through direct or indirect animal contact would be expected to spread rapidly in the study system. However, the observed heterogeneity in between-farm contact may present opportunities for interventions to be targeted to particular herds to limit infectious disease spread.

## Highlights

• Degree of farm contact and the distances between farms were negatively correlated.
• Disease control and surveillance to consider between farms contacts in the villages.
• Heterogeneities in between farm contact may limit infectious disease spread.

## 1 Introduction

In livestock production systems that rely on the use of common grazing areas, direct contacts between animals from different farms can be expected to be common during grazing and watering. They may also occur at central vaccination or tick control points, at livestock markets, or through sharing of bulls for breeding or ploughing. Indirect contacts may also occur through sharing of equipment or movement of people between farms (Webb, 2005). Such contacts may link farms to form networks and act as routes through which infectious agents can spread (Gupta et al., 1989).

Network analysis has been used widely to study the social networks underlying the spread of a range of infectious diseases (Jolly et al., 2001). The need for a better understanding of the contact networks underlying farm populations has been shown by historical disease epidemics, including the 2001 foot and mouth disease (FMD) epidemic in Great Britain (GB). In that instance, lack of information on between-farm contact structures hindered scientists from developing models to predict disease spread (Woolhouse and Donaldson, 2001), and suggested that livestock contact networks needed to be better understood before disease outbreaks occur.

Limited research has been done on between-farm contact networks in developing countries. These are likely to be complicated by the continuous adaptation of livestock keepers to variable environmental and socioeconomic conditions in these settings (Waret-Szkuta et al., 2011). Description of the contact structures of livestock in countries without registered animal movement is also a major challenge. One of the first studies investigating animal movements to be conducted in sub-Saharan Africa examined contacts occurring at common water and grazing points around selected villages in the highlands of Ethiopia using an interview-based approach (Waret-Szkuta et al., 2011). The authors reported high levels of variability in the contact structure between villages. Understanding the factors that influence this variability, and the extent to which individual animals and herds are linked to a community of herds, may be important for understanding the epidemiology of infectious diseases and for designing control and prevention measures. Contact networks are made up of nodes which have varying degrees of connection with other nodes in the network. Characteristics of these networks may have important implications on the transmission of infectious diseases within a population (Lloyd and May, 2001) and on the efficacy of vaccination programmes (Zanette and Kuperman, 2002). Identifying highly connected nodes, be they individuals, herds or whole areas, may therefore allow the efficient targeting of limited resources to prevent or control infectious disease outbreaks.

Social network analysis (SNA) is increasingly used in veterinary epidemiology to describe the topology of direct and indirect contacts in livestock populations (Dube et al., 2009). When these network links are associated with known risk factors for disease transmission, the impact of the network structure on the potential routes of transmission of infectious diseases can be hypothesized (Waret-Szkuta et al., 2011). The aim of the study was to use SNA to describe contact types and their structure at the village level in a smallholder farming system in western Kenya and to consider the implications of the networks identified on infectious disease transmission in this setting.

## 2 Methods

### 2.1 Study area

**Fig. 1:**
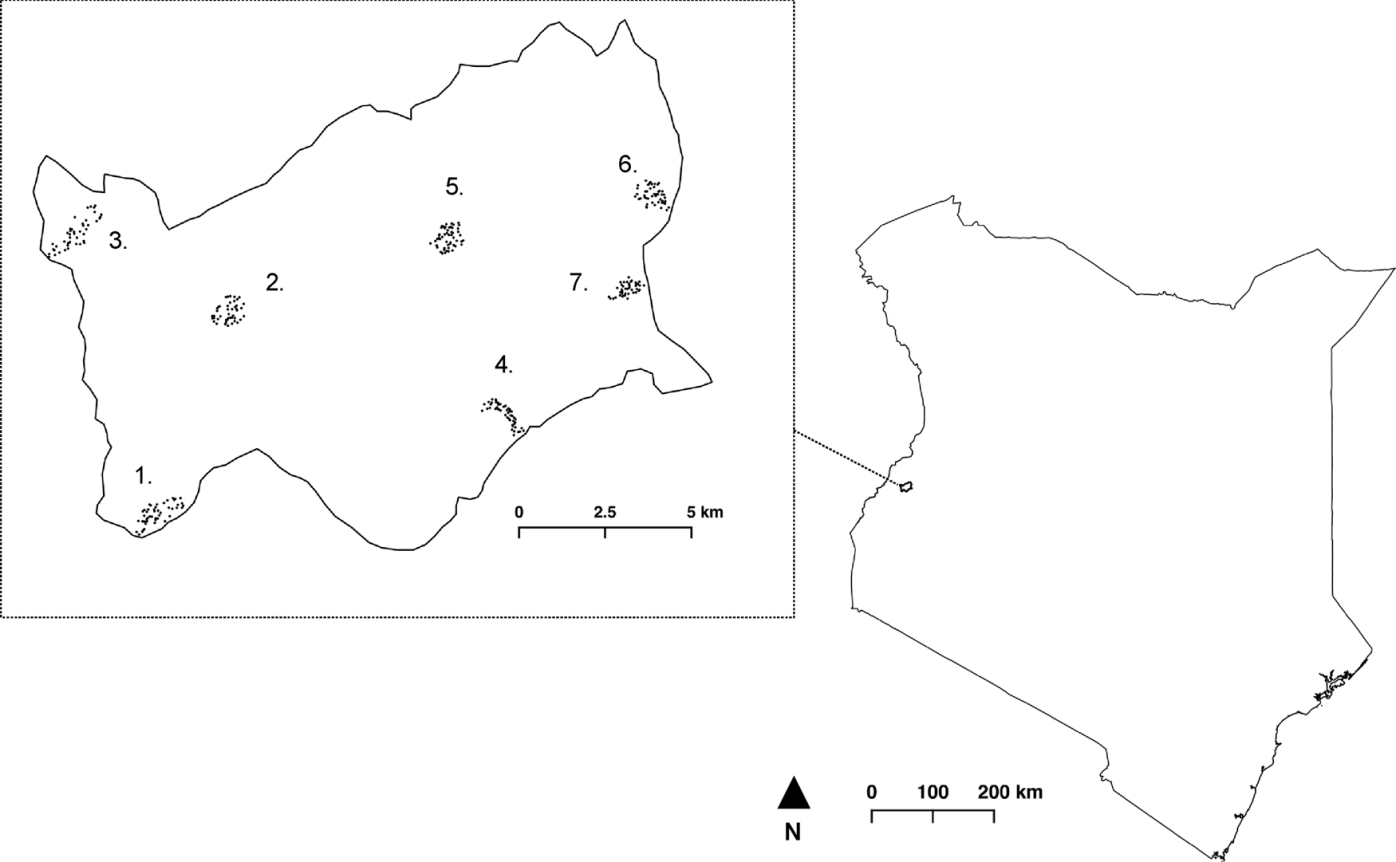
Location of Kimilili district and cattle keeping households in study villages in Kenya (1 = Chebukwabi; 2 = Malaha; 3 = Kibunde; 4 = Namunyiri; 5 = Lutonyi; 6 = Lurare and 7 = Sango).

The study was undertaken in Kimilili district in Bungoma County, Kenya. Bungoma County has a population of 333,532 head of cattle and 1,076,367 households (Kenya National Bureau of Statistics, 2009). The area is characterized by a mixed smallholder farming system, in which livestock production is integrated with crop production. Cattle in the county are used for small-scale dairy and meat production, as well as for traction and as source of income. The average cattle herd size in the area is 5 cattle per farm (Fèvre et al., 2017) with the majority of these being local zebu cattle, and smaller numbers of exotic dairy and cross breeds. Cattle are reared under free-grazing, tethering, or zero-grazing and depend on natural pastures, fodder crops and agricultural by-products as their main feed source (Mudavadi et al., 2001). The agro-ecological zones of Bungoma County range from Upper Midland Zone 1 to Lower Midland Zone 4 with a total area of 1684 sq km. The study site is shown in Fig. 1.

### 2.2 Sampling method

A full list of all the villages in Kimilili district was obtained from the office of the local administrator (District Commissioner’s office). Seven villages out of 43 were selected using random number generation (Chebukwabi (village 1), Kibunde (village 2), Lutonyi (village 3), Malaha (village 4), Namunyiri (village 5), Lurare (village 6) and Sango (village 7)). Recruitment involved a visit to the assistant chief for the sub-location in which the village fell. The study approach and its expected outcomes were explained, and permission to work within each village was obtained.

### 2.3 Data collection

A census of all cattle keeping households within each selected village was undertaken in consultation with a village elder. For all households keeping cattle, the study was explained and the participation of the household head and main cattle keeper was obtained. Consenting farmers had their full names collected and a face-forward portrait photograph of the head of the household (and/or other individual with responsibility for cattle) was captured using a digital camera (CANON, Focal length 7.4–44.4, Japan). Farmers provided oral consent for participation following full explanation of the study and the photographs were deleted upon study completion.

A day after village recruitment, each consenting household was re-visited and a structured photo-elicitation interview was performed. For this, the digital portrait photographs of all consenting farmers within the same village were shown to the household head and any person within the household who had responsibility for cattle management (those people who take cattle out for grazing or to water points). At the same time, the full name of the person(s) in the photograph was said out loud. Interviewees were asked about any contacts between their own animals and those owned by the person(s) in the photograph during the past 4 weeks. Specific contacts were separated into those occurring during grazing, watering or within or at the boundary of the homestead (defined as the collection of households occupied by a single extended family). Contacts were defined as either (1) herds coming into direct body mixing with other herds or (2) indirect contact such as grazing in the same field but no physical body contact. At the same time, farmers were asked to verbally recall between-herd contacts with animals owned by the individual(s) in the photograph that involved the use of shared bulls for breeding and ploughing in the past 12 months. Data on the number of cattle owned, herd composition and management practices were also collected.

Co-ordinates were collected from a central point within the homestead using a Global Positioning System (GPS) hand held receiver (eTrex, GARMIN® International Inc. Kansas, USA). All data were collected between November and December 2013, during the period of the “short rains”.

### 2.4 Ethical approval

Ethical approval for data collection from human subjects was granted by the Kenya Medical Research Institute (KEMRI) Ethical Review Committee (SSC No.1701).

### 2.5 Data management and analysis

Data were entered into Microsoft Excel^®^ 2003 (Microsoft Corporation, USA). Network analysis was performed for direct and indirect contacts in the past four weeks, breeding contacts in the past 12 months and overall farm contacts in the past 12 months for each village using UCINET 6.182 (Borgatti et al., 2002). The four week contact network considered only direct and indirect contacts occurring at grazing, watering or the boundary of the homestead in the past four weeks. Overall contacts were defined as any direct or indirect contacts between animals at grazing, watering and/or at the boundary of the homestead in the previous four weeks and/or breeding/ploughing contact in the previous 12 months. We assumed that the four week grazing, watering and boundary contacts were broadly representative of grazing and watering contacts over the previous 12 months and therefore that the overall network represents all between-herd contacts over the course of a year. Breeding contact was defined as presence of a mature non-castrated male having sexual contact with a mature cow from another farm outside the homestead and vice versa.

Network density (the proportion of all possible contacts, or links, that were actually present) and number of isolates (cattle keeping households, or nodes, not connected to any other) were calculated for each network. The degree for each node (number of links incident to a given node) was normalized (degree of the node divided by the number of nodes in the network) to allow comparison between villages. The mean normalized degree and average geodesic distance (mean number of links in the shortest path between all reachable pairs of nodes) was also extracted for each village and each contact type. The clustering coefficient (the sum of the proportion of nodes that are directly connected to another node) and degree centralization (measure of “importance” of the individual nodes) were also extracted, as was the network diameter (the longest geodesic distance between any pair of farms in a network). Centrality measures included betweenness centrality, which is the frequency with which a node falls between a pair of other nodes on the geodesic path connecting them and closeness centrality, which is the distance from one node to all others in the network. The normalized betweenness (RBci) and normalized closeness, which allow for comparison of values of nodes from different networks (Gould, 1987), were also calculated.

A bootstrap paired *t*-test was used to test for differences between described networks in each village. A total of 5000 random permutations were run per test to meet assumptions of independence and random sampling (Hanneman and Riddle, 2005). Dyadic quadratic assignment procedure (QAP) correlation was used to calculate the correlation between contact network matrices for grazing and water contacts and breeding and ploughing contacts using a Pearson correlation coefficient based on 10,000 random permutations (Hanneman and Riddle, 2005). QAP was also used to assess the correlation between the presence/absence of contact between farms and distance (in kilometres) between them.

## 3 Results

### 3.1 Questionnaire results

A total of 329 farms participated in seven villages with an average of 47 farmers per village (range 43–53). All cattle keeping households in the study villages agreed to participate, with all heads and/or other individuals with responsibility for cattle consenting to having their photographs taken. The proportion of farms that practiced extensive grazing management during the dry season was 14.3% (95% CI 10.9–18.5) and the number dropped slightly to 12.8% (95% CI 9.6–16.9) during the rainy season. The majority of farmers practiced semi-intensive grazing management during both the dry and rainy seasons (85.4% and 87.2% respectively). This involved tethering their cattle for the majority of the day and then grazing them after attending to other domestic chores or when children were back from school. The proportion of farmers that reported taking their animals outside their farms for watering was 38.9% (95% CI 33.8–44.3) during the rainy season, with 94.4% of these indicating rivers as the main source of water. The proportion of farmers watering cattle outside the farm increased to 42.9% (95% CI 37.6–48.3) during the dry season. The proportion of farmers that reported taking their animals outside their own farms to pastures during the rainy season was 53.2% (95% CI 47.8–58.6) and the number increased to 61.7% (95% CI 56.5–67.0) during the dry season. The proportion of farmers that introduced new animals into their herds during the last 12 months was 31.3% (95% CI 26.5–36.6) while 35.9% (95% CI 30.8–41.2) had sold at least one of their animals in the past 12 months. The average number of animals sold was 1.9 (range 1–9) while the average number introduced was 1.8 (range 1–10). A small number of farms, which could be considered as being commercial livestock traders reported buying (6.4%) and selling (5.6%) ten animals and above. Movement of cattle within study villages through sales was low with only 3.3% and 5.3% of farms reporting buying and selling, respectively, between households in the same village.

### 3.2 Network analysis

Summaries of network statistics for overall contacts are presented in Table 1 for the seven villages. The average density of overall farm contacts was highest in village 1 (14%) and lowest in village 5, with 89 undirected ties out of the maximum possible 925 undirected links (9.6%). Degree centralization was highest in village 7 (36.8%), with a small number of nodes dominating the network (high centrality). All overall networks had one component with slightly varying sizes and villages 1, 2 and 7 were completely connected (no isolates). The remaining villages had a small number of isolates (village 5 and village 6 (1), village 3 (2) and village 4 (3)). The normalized betweenness centrality was highest in village 5 (5.7) and lowest in villages 6 (3.0) and 4 (3.0). Normalized closeness centrality, which focuses on the average distance of an actor to all others in the network, was highest in village 1 (41.7) and lowest in village 4 (17.9). The average geodesic distance was highest in village 5 (3.4) and lowest in village 6 (2.4).

**Table 1:** Summary of parameters of the overall contact matrices including combined grazing/watering contacts in the previous four weeks and/or breeding/ploughing contacts in the previous 12 months.

Village	1	2	3	4	5	6	7
No. nodes	44	48	45	53	43	48	48
Density (undirected) (%)	14.0	11.8	12.9	10.6	9.6	13.1	11.5
Normalized degree variance	67.6	42.2	51.2	41.5	57.2	69.1	50.0
Degree centralization (%)	29.2	21.0	17.4	25.0	34.8	21.8	36.8
Diameter	6	6	6	6	10	5	6
No. reachable pairs	946	1128	903	1225	861	1081	1128
% pairs reached	100	100	91.2	88.9	95.3	95.8	100
Number of components	1	1	3	4	2	2	1
Component size	44	48	43,1[2]	50, 1[3]	42,1	47,1	48
Av. geodesic distance	2.5	2.6	2.7	2.7	3.4	2.4	2.6
Normalized closeness	41.7	38.8	21.8	17.9	23.8	29.8	39.6
Normalized betweenness	3.5	3.5	3.6	3.0	5.7	3.0	3.4
Coefficient of Variation	1.7	1.8	1.8	1.6	1.3	1.6	1.6

^1^Numbers in square brackets represent the number of repeats of that value.

**Fig. 2:**
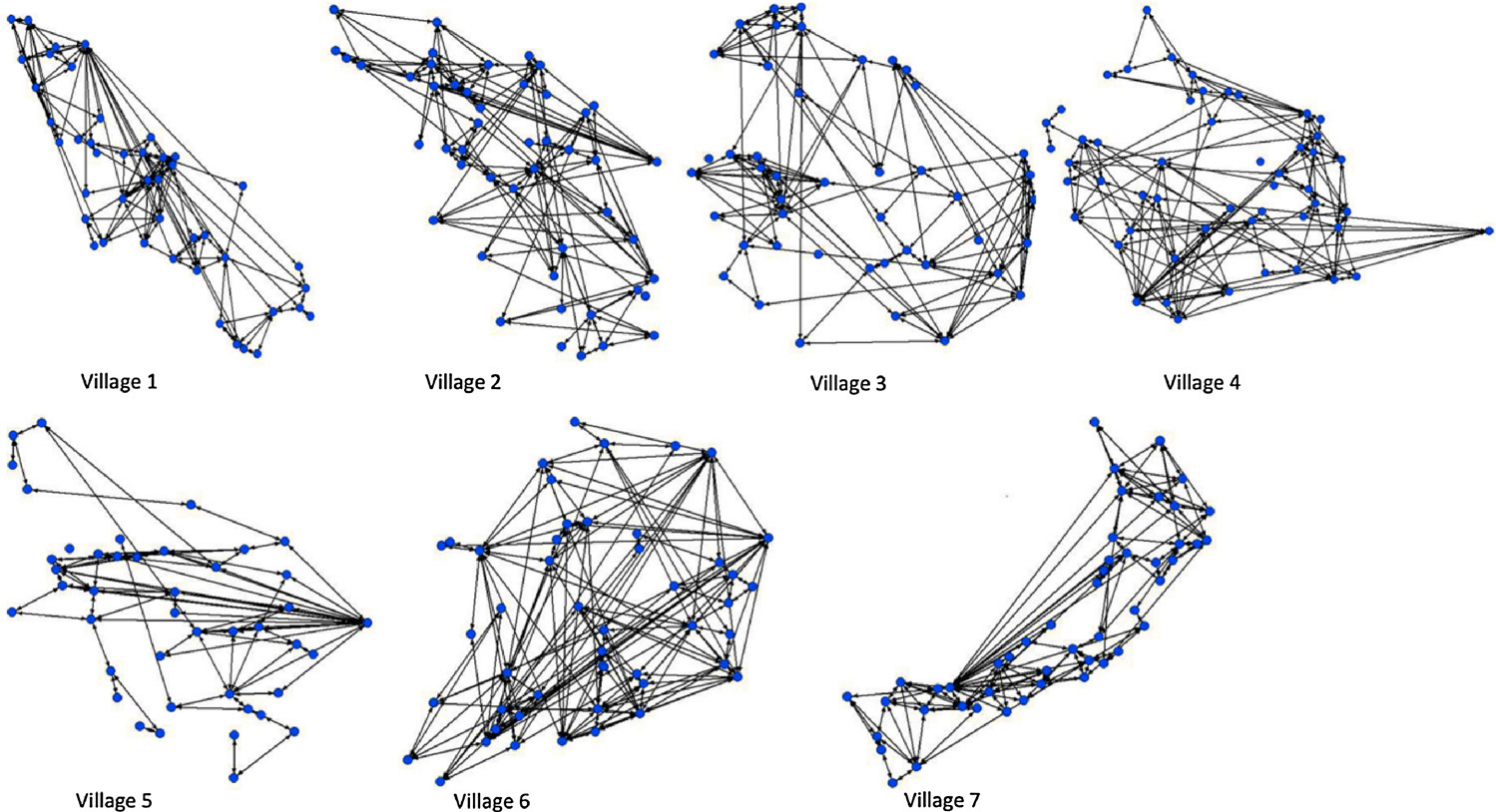
Overall contact networks (at grazing and water in the past 4 weeks and breeding/ploughing in the past 12 months). Nodes represent farms and lines respresent contact between farms. Household position represents the relative geographic location of the household in each village.

The graphs in Fig. 2 give overall contacts for cattle keeping households in study villages.

**Fig. 3:**
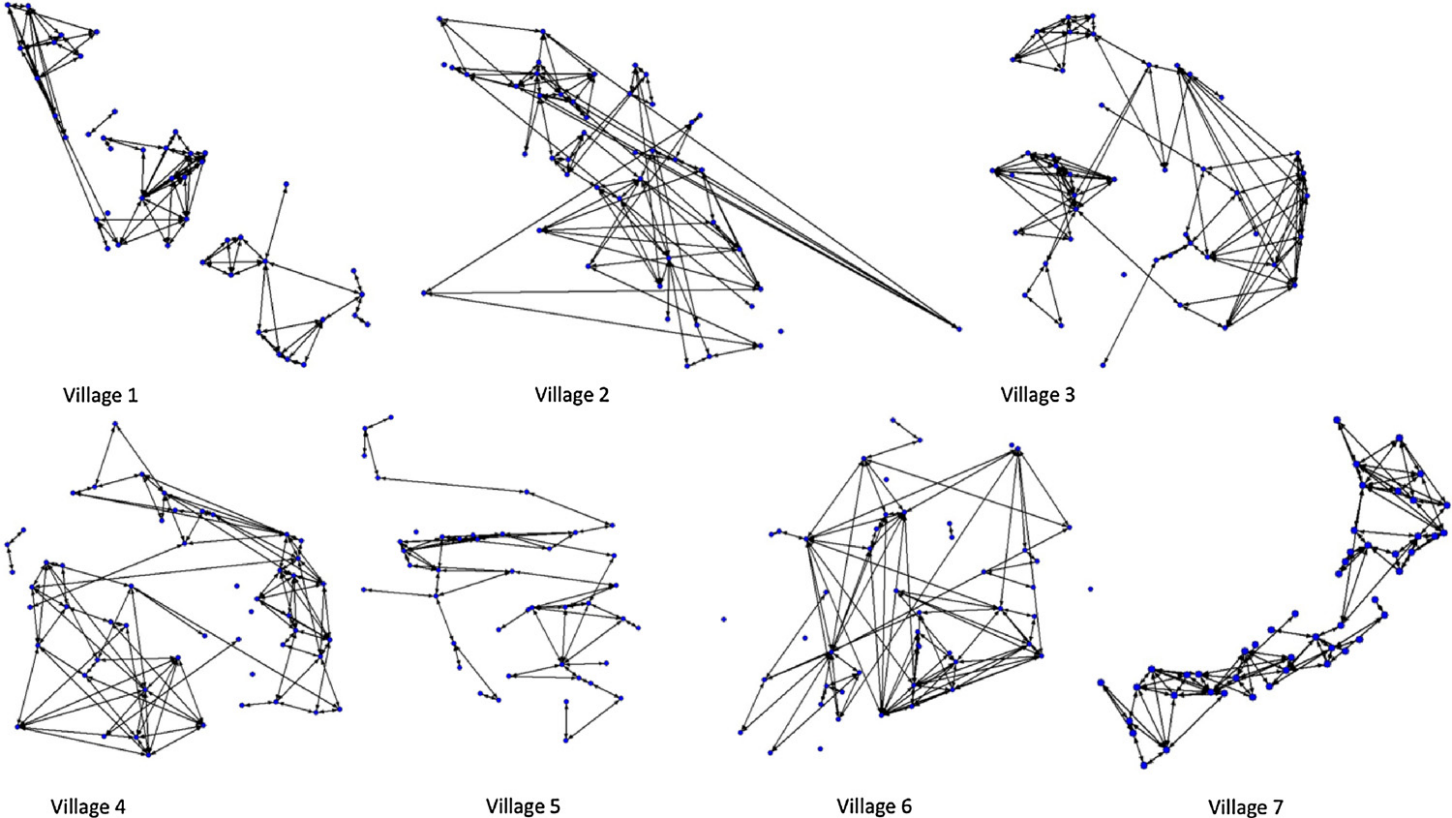
Contacts at grazing and water over the past 4 weeks. Nodes represent farms and lines represent contact between farms. Household position represents the relative geographic location of the household in each village.

The four week contact (through grazing and water direct or indirect contact) network is shown in Fig. 3, with parameters for these networks presented in Table 2. The normalized degree variances and network centralizations for the four week contacts were highest in villages 1, 3 and 6: an indication of greater heterogeneity in the farm contacts in these villages. These networks demonstrate high levels of connectivity, with between 46 and 100% of households in each study village linked through direct or indirect contact during grazing or watering.

**Table 2:** Summary of network parameters for contact at common water points, farm boundary and open field grazing contacts in the past four weeks.

Village	1	2	3	4	5	6	7
No. nodes	44	48	45	53	43	48	48
Density (undirected) (%)	10.0	8.7	11.0	7.6	7.0	8.2	9.3
Normalized degree variance	33.6	25.2	39.0	22.9	24.4	49.7	21.4
Degree centralization (%)	16.3	15.4	14.6	10.1	10.2	18.1	10.3
% pairs reached	45.6	87.8	91.2	75.3	82.1	69.2	100
Number of components	4	4	3	6	5	8	1
Component size	26, 15, 1[2]	45, 1[3]	43, 1[2]	46, 3, 1[4]	39, 1[4]	40, 2, 1[6]	48
Av. geodesic distance	2.6	3.5	4.1	3.5	4.9	3.1	4.4
Normalized closeness	4.3	15.9	17.1	9.3	11.9	9.0	23.7
Normalized betweenness	1.7	4.7	6.5	3.7	7.8	3.2	7.3

^1^Numbers in square brackets represent the number of repeats of that value.

**Fig. 4:**
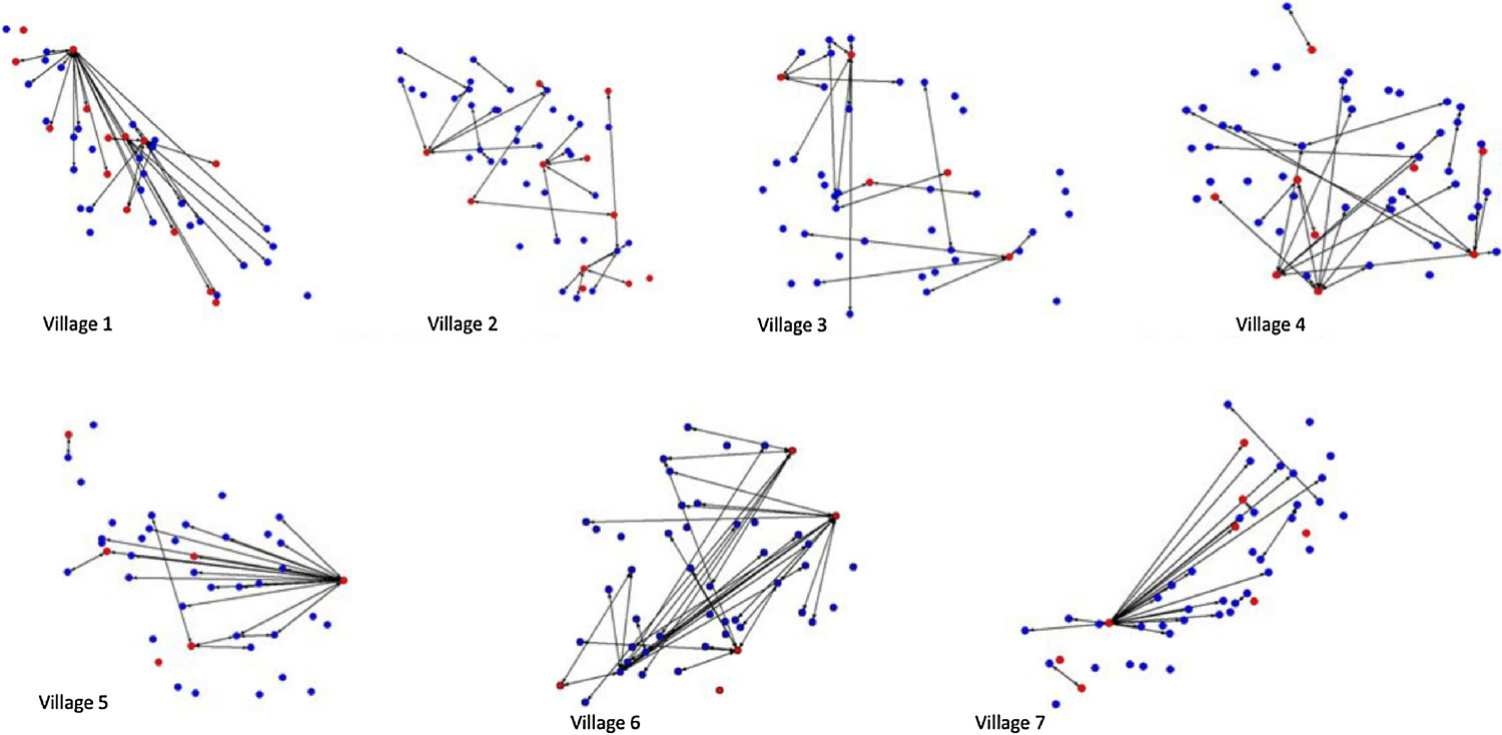
Breeding contact over the past 12 months network; Red nodes represent farms with bulls, blue farms with cows and lines represent contact between farms. Household position represents the relative geographic location of the household in each village (For interpretation of the references to colour in this figure legend, the reader is referred to the web version of this article).

Summaries for the average density and network centralization for the breeding network are presented in Table 3, and the graphs of the breeding networks in each village in Fig. 4. Network centralization and normalized degree variance were highest in villages 1, 5 and 7, indicating higher levels of variability and diversity of breeding contacts in these villages. These networks demonstrate varying levels of connectivity, with between 7.6 and 59% of farms in each study village linked through sharing of breeding bulls.

**Table 3:** Summary of network parameters for breeding contacts in the past 12 months.

Village	1	2	3	4	5	6	7
No. nodes	44	48	45	53	43	48	48
Density (undirected) (%)	3.0	2.2	1.9	2.0	2.7	3.3	2.3
Normalized degree variance	35.5	7.2	7.8	7.5	41.7	27.2	35.6
Degree centralization (%)	35.9	8.8	12.3	12.0	42.2	25.4	42.0
% pairs reached	19.8	7.6	7.7	8.0	32.4	59.0	19.2
Number of components	16	23	26	26	20	12	22
Component size	17, 10, 4, 13[1]	9, 8, 6, 2[3], 2, 1[17]	11, 2[5], 2, 1[22]	10, 10, 6, 3, 2[2] 1[20]	23, 2, 1[18]	37, 1[11]	21, 3, 4[2], 16[1]
Av. geodesic distance	1.9	2.0	2.4	2.0	2.3	3.7	1.9
Normalized closeness	3.2	2.4	2.6	2.2	4.4	7.1	3.0
Normalized betweenness	0.4	0.2	0.2	0.2	0.9	3.4	0.4

^1^Numbers in square brackets represent the number of repeats of that value.

Using adjacency matrices describing the sharing of farm attributes, there was evidence that farm distances and breeding contacts were weakly negatively correlated in the majority of study villages (Table 4). There was evidence of a weak positive correlation in village 6 (Table 4). Between farm distances were negatively correlated with overall farm contacts and contacts in the past 4 weeks in all of the study villages (Table 4).

**Table 4:** Pearson correlation between farm distance and overall between-farm contacts (including breeding movements and grazing and watering contacts in the past 4 weeks).

Village	Breeding contact		Grazing/watering contact in past 4 weeks		Overall contact	
	Pearson Correlation	P-value	Pearson Correlation	P-value	Pearson Correlation	P-value
1	0.0	0.31	−0.4	<0.001	−0.4	<0.001
2	−0.2	<0.001	−0.3	<0.001	−0.3	<0.001
3	−0.1	0.009	−0.4	<0.001	−0.4	<0.001
4	−0.1	0.002	−0.4	<0.001	−0.3	<0.001
5	−0.1	0.13	−0.3	<0.001	−0.3	<0.001
6	0.1	0.02	−0.3	<0.001	−0.2	<0.001
7	−0.1	0.07	−0.4	<0.001	−0.3	<0.001

The mean degree of overall contact was higher for farmers who took their cattle to common water points than those that watered livestock within their farms in all villages (Table 5). Farms where semi-intensive grazing management was practiced also tended to have a lower mean degree of overall contact (Table 6).

**Table 5:** Comparison of the mean degree of overall contact for farms where cattle were taken to common water points and those watered at home.

Village	Mean 1 (Water at home)	Mean 2 (Common water points)	Difference in Mean degree	p-value
1	10.3	16.3	−6.0	0.016
2	10.6	14.0	−3.4	0.085
3	10.0	20.7	−10.7	<0.001
4	7.0	14.8	−7.8	0.002
5	10.0	14.3	−4.3	0.12
6	9.4	12.7	−3.3	0.42
7	9.3	13.2	−3.9	0.046

**Table 6:** Comparison of overall mean degrees of contacts for farms practicing semi-intensive and extensive grazing management.

Village	Mean 1 (Semi-intensive grazing management)	Mean 2 (Extensive grazing management)	Difference in mean degree	Two-Tailed t-test
1	13.5	20.2	−6.7	0.19
3	11.5	20.1	−8.6	0.005
4	10.9	22.2	−11.2	<0.001
5	10.5	11.2	−0.6	0.87
6	9.6	10.7	−1.1	0.82
7	9.6	15.7	−6.1	0.003

^*^Village 2 had farms only practicing semi-intensive grazing management.

Results from the Pearson correlation test provided evidence that breeding and ploughing networks were correlated in four villages (Table 7), water and extensive grazing networks were correlated in five villages and water and farm boundaries grazing networks were also correlated in five villages.

**Table 7:** Correlation between breeding and ploughing networks, water and extensive grazing networks, water and farm boundaries grazing networks.

Village	Breeding and ploughing networks		Water and extensive grazing networks		Water and farm boundaries grazing networks	
	Pearson Correlation	p-value	Pearson Correlation	p-value	Pearson Correlation	p-value
1	0.0	0.49	0.2	0.001	0.3	<0.001
2	0.2	0.001	0.1	0.06	0.1	0.009
3	0.1	0.031	0.7	<0.001	0.1	<0.001
4	0.1	0.009	0.1	0.003	0.0	0.237
5	0.1	0.15	0.1	0.14	0.2	0.004
6	0.1	0.15	0.6	<0.001	0.1	0.028
7	0.4	<0.001	0.8	<0.001	0.0	0.52

## 4 Discussion

The aim of the study was to assess the types and frequency of contacts between herds in a mixed farming area of western Kenya, and to consider the implications for disease transmission. We find that all or virtually all households in a random selection of villages could be connected (directly or indirectly) through contacts occurring during grazing or watering and/or through movements of animals for breeding or ploughing over the past 12 months. Moreover, in almost all villages, the majority of households could be connected (directly or indirectly) through contacts occurring during grazing and watering in the past four weeks. We did not explicitly model disease transmission in these networks but, given these findings, consider that the spread of pathogens that are transmitted through direct or indirect contact could be rapid and widespread following introduction. These findings have important disease control implications, and suggest that the control of trans-boundary diseases, such as foot and mouth disease, will require disease control interventions that encompass whole communities rather than individual herds in the study area.

We also observed heterogeneity in the contact networks at both the household and village level. This finding is similar to results from a study on herd contact structure in Ethiopia (Waret-Szkuta et al., 2011). We did not include enough villages to explore factors influencing contact variability at the village level, but we expect that these differences could be explained in part by differences in the environmental context and farmer composition of each. For example, the absence of a river passing through Malaha and Namunyiri (Villages 4 and 5) meant that most farmers provided water to their livestock in their homes. Not unexpectedly, we identified that farms where livestock were taken to common water points tended to have more contacts. This may explain the comparatively lower network density in these two villages. Similarly, most farmers practiced semi-intensive grazing management in Chebukwabi (Village 1), which may be due to a lack of dedicated communal grazing areas in this village (although this was not recorded). There was a general trend for reduced contact between cattle owned by those farmers practicing semi-intensive grazing compared to extensive grazing. This may explain why Chebukwabi had the highest levels of network fragmentation (and lowest proportion of potential pairs reached). The heterogeneities observed between study villages suggest introduction of infectious diseases could have variable rates of spread in each (Albert et al., 2000). Future work with a larger number of study sites could examine the association between levels of within village connectivity and the contextual and compositional characteristics of study communities. This could assist livestock disease surveillance activities by identifying those villages in which the spread of disease is most likely.

Overall, contacts in study villages were negatively correlated with between-farm distance. Hence, farms that were closer together geographically were more likely to be linked through livestock contacts compared to farms that were farther apart. This effect was observed for grazing and watering contacts over the previous four weeks as well as for overall contacts and, to a lesser extent, for breeding contacts. This observation reflects findings by Mahmood et al. (2010) on the influence of geographical distance on contact network formation. We used geodesic distance in this study since all the farmers are on foot and tend to graze around households or nearby communal grazing areas. Other mechanisms of disease spread exist, such as through fomites like vehicles and humans (Allerson et al., 2013; Mansley et al., 2011), that may make measures such as road distances between farms a more appropriate measure of between-farm distance in other settings.

This study has a number of potential limitations that need to be considered. We examined only those contacts occurring between farms in the same village. However, most farmers indicated sharing of breeding/ploughing bulls with households in other villages, and grazing and common water points contacts with animals in neighboring villages. Whilst villages tend to be geographically separated in the study area, the focus only on within-village contacts means that between-farm contacts are likely to have been underestimated, particularly for those households on the periphery of each village. Because of a lack of official records on livestock movement, we had to rely on farmer recall, which could be expected to underestimate the number of contacts. We sought to improve the accuracy of this method by limiting discussion of grazing and watering contacts (which occur more frequently and are less likely to be remembered than contacts associated with breeding and ploughing) to the past four weeks and through the use of a photo-elicitation technique. We believe the photo-elicitation approach improved farmer recall, as we found farmers were often only able to remember actual or nick names of a limited number of their neighbours, or not able to remember their names at all. The use of photo elicitation process is expected to have increased the capture of between-farm contacts, with participants likely to have been reminded of contacts that may have been forgotten using only an interview based approach. While the approach used represents a relatively rapid method to derive between-farm contact data, we cannot assess the accuracy of the method in estimating all within-village contacts. Future work could compare the performance of this approach to more time consuming but presumably more accurate approaches, such as researcher-based observation of contacts (DeWalt and DeWalt, 2002). This study was conducted during the wet season, when pastures and water are readily available and therefore when farmers may not move their livestock far away from their homesteads. While there was no significant difference in the proportion of farmers reporting grazing their animals or accessing water off their farm between dry and wet season, we would expect contact rates to increase during the dry season when farmers are likely to have to travel farther for grazing and water. It is also important to note that this study was undertaken during the period when most crops (which are typically grown around the homestead) had been harvested. This may encourage free or tethered grazing in the area closer to households, further reducing the number of grazing contacts. These limitations, combined with the lack of between-village contact estimation, and the likelihood of some degree of omission error, mean it is likely that the level of between-farm contacts described, and therefore the expected potential for disease spread, is conservative

Animal mixing at watering and grazing points has been identified as a key factor for transmission of diseases such as foot and mouth disease (FMD) and peste des petits ruminant (PPR) (Lefevre et al., 2003). We observe that farmers with high levels of contact in the grazing network tended to also have high levels of contact in the watering network, increasing their herd’s risk of acquiring infection and spreading it. Promotion of the provision of water to cattle within homesteads and/or wider adoption of semi-intensive management practices would be expected to reduce between-herd contacts within study villages. Clearly, the promotion of such measures would need to be made in conjunction with an assessment of local grazing and water availability: most farmers are likely to be highly reliant on access to communal water and grazing areas for their livelihoods. In the study villages, there tended to be a small number of farms with breeding bulls that were used widely for breeding. Many farmers castrate bulls to make them docile so that they can be used for draught work and for transport, and the resulting low numbers of breeding bulls increases the chance of widespread inbreeding. Partner number is well-known to be linked to a higher risk of infection with sexually transmitted diseases (Ghani et al., 1997; Gupta et al., 1989). The presence of a small number of breeding bulls making sexual contacts frequently may therefore also have important implications for transmission and persistence of sexually transmissible diseases, such as brucellosis, vibriosis and trichomoniasis in the population under study. Identifying and targeting such animals for interventions, such as vaccination or test and treatment, could therefore contribute to reducing the transmission of infectious disease within study communities.

## 5 Conclusion

We observed high levels of between-farm contacts in a random selection of villages in a rural, mixed farming area of western Kenya. The high observed inter-connectedness is likely to facilitate within-village transmission of infectious diseases, and suggests considering the village as a single interacting “herd” may be useful in the approach to surveillance and control in this setting. We also observed that some farms had higher levels of contact with others in the same village as a result of sharing breeding bulls, use of communal watering and grazing points, and these farms may represent targets for control to reduce spread of infectious disease within a single village.

## Declaration of interest

None.

## Funding sources

This project was funded by The Wellcome Trust, grant number 085308 and The Biotechnology and Biological Sciences Research Council, Department for International Development, the Economic & Social Research Council, the Medical Research Council, the Natural Environment Research Council and the Defence Science & Technology Laboratory, under the Zoonoses and Emerging Livestock Systems (ZELS) programme, grant reference BB/L019019/1. WAdeG was funded by a UK BBSRC DTG. This work also received support from the CGIAR Research Program on Agriculture for Nutrition and Health (A4NH), led by the International Food Policy Research Institute (IFPRI). We also acknowledge the CGIAR Fund Donors (https://www.cgiar.org/funders). The funders had no role in study design, data collection and analysis, decision to publish, or preparation of the manuscript.
